# A New Children's Hospital

**Published:** 1918-01-05

**Authors:** 


### A New Children's Hospital.

Christmas witnessed the removal of the Birmingham Children's Hospital from the old quarters in Broad Street to the new in Ladywood Road. The new building, which was designed by the late Mr. F. W. Martin, and built by Messrs. J. Barnsley and Sons, is the city's memorial to King Edward. The memorial stone was laid on April 12, 1913, when the memorial fund had reached a total of £33,000. It will be remembered that the plumbers' strike caused an early delay in the building. Later came the war, but at last the initial building, to which an out-patient department will be added, is complete. On a T plan the administrative block is a comprehensive building, while the wards are planned in two three-storey pavilions inclined at an angle towards each other. (See The Hospital, June 21, 1913, p. 367.) Each ward unit possesses twenty-five beds, has a flat roof, and its own theatre. A fresh appeal is about to be issued to meet the excess on the estimates which the war and other causes have occasioned.

